# Symptom-specific effects of counselling for depression compared to cognitive–behavioural therapy

**DOI:** 10.1136/bmjment-2022-300621

**Published:** 2023-02-15

**Authors:** Ciarán O'Driscoll, Joshua E J Buckman, Rob Saunders, Sarah Ellard, Syed Ali Naqvi, Satwant Singh, Jon Wheatley, Stephen Pilling

**Affiliations:** 1 CORE Data Lab, Centre for Outcomes Research and Effectiveness, Research Department of Clinical, Educational and Health Psychology, University College London, London, UK; 2 iCope—Camden & Islington Psychological Therapies Services, Camden and Islington NHS Foundation Trust, London, UK; 3 Whittington Health NHS Trust, London, UK; 4 North East London NHS Foundation Trust, Rainham, Havering, UK; 5 Talk Changes: City and Hackney IAPT Service, Homerton University Hospital NHS Foundation Trust, London, UK; 6 Camden & Islington NHS Foundation Trust, London, UK

**Keywords:** Depression & mood disorders, Adult psychiatry

## Abstract

**Background:**

Cognitive–behavioural therapy (CBT) and counselling for depression (CfD) are recommended first-line treatments for depression. While they approach change differently, there is little understanding of the impact those approaches have on change during treatment.

**Objectives:**

This study aimed to identify whether CBT and CfD target different symptoms and explore the implications of modelling choices when quantifying change during treatment.

**Methods:**

Symptom-specific effects of treatment were identified using moderated network modelling. This was a retrospective cohort study of 12 756 individuals who received CBT or CfD for depression in primary/community care psychological therapy services in England. Change was modelled several ways within the whole sample and a propensity score matched sample (n=3446).

**Findings:**

CBT for depression directly affected excessive worry, trouble relaxing and apprehensive expectation and had a stronger influence on changes between suicidal ideation and concentration. CfD had a stronger direct influence on thoughts of being a failure and on the associated change between being an easily annoyed and apprehensive of expectation. There were inconsistencies when modelling change using the first and second appointments as the baseline. Residual score models produced more conservative findings than models using difference scores.

**Conclusions:**

CfD and CBT for depression have differential effects on symptoms demonstrating specific mechanisms of change.

**Clinical implications:**

CBT was uniquely associated with changes in symptoms associated with anxiety and may be better suited to those with anxiety symptoms comorbid to their depression. When assessing change, the baseline should be the first therapy session, not the pretreatment assessment. Residual change scores should be preferred over difference score methods.

**WHAT IS ALREADY KNOWN ON THIS TOPIC**

- Cognitive–behavioural therapy (CBT) and counselling for depression are recommended first-line treatments for depression and are considered equally effective on average. However, little is known about how change comes about.

**WHAT THIS STUDY ADDS**

- This study investigates symptom-specific effects and identifies specific symptoms and symptom interactions associated with each intervention. In addition, it highlights methodological considerations when modelling change.

**HOW THIS STUDY MIGHT AFFECT RESEARCH, PRACTICE OR POLICY**

- CBT was uniquely associated with changes in symptoms associated with anxiety so may be better suited to those with anxiety symptoms comorbid to their depression.

## Background

There is a strong preference among patients for psychological therapies over antidepressant medications.^1^ Cognitive–behavioural therapy (CBT) and counselling for depression (CfD) are among the most used psychological therapies for depression, both are efficacious and recommended as first-line treatments for depression.^2^ They are equally effective on average, but many patients do not experience symptomatic improvement with these treatments.^3^ There is some evidence that outcomes can be improved by identifying for whom each type of treatment is most likely to be beneficial.^4^ However, precision mental healthcare is hampered by a lack of understanding of how the individual treatments bring about symptomatic improvements,^5^ and issues of measurement that affect the accuracy and utility of precision models.^6^

The symptom experiences of people with depression are heterogeneous^7^ with evidence of differential treatment effects on specific symptoms.^8 9^ During psychotherapy, change in one symptom is highly dependent on other symptoms^10^ and effects of a treatment when controlling for the influence of all other symptoms are likely to be small. Modelling the direct influence of treatments on symptom change may elucidate unique differences between treatments, informing how treatments work and thus the potential suitability of a given treatment for an individual based on their pretreatment characteristics.

CfD aims to engender change by exploring the emotional meaning associated with experiences and developing alternative ways of understanding these experiences to inform a new self-concept.^11^ CBT for depression, on the other hand, aims to bring about change through cognitive processes (eg, challenging negative automatic thoughts) and behavioural processes (eg, reduced avoidance and balancing activities).^12^ A recent clinical trial demonstrated the non-inferiority of CfD at 6 months but inferiority to CBT at 12 months,^13^ while analyses of routine clinical data suggest that at the aggregate level, outcomes are comparable.^14^ Two studies have highlighted the potential for pretreatment data to be used to stratify patients into groups that are more likely to benefit from one of these types of treatment than from the other.^15 16^ One was an exploratory study, and the other had only a small sample receiving CfD. Those studies were not able to investigate the differential effects of the treatments on symptoms so could not elucidate mechanisms. They also used outcomes based on pre–post treatment change which can introduce a high degree of bias,^17^ the first of which was a pretreatment assessment occurring sometime before treatment started and may not be an appropriate baseline. The implications of different methods of calculating change within clinical trials have been investigated thoroughly (see online supplemental eMethod). Capturing the nuance in symptom profiles and illustrating how best to overcome the issues of bias in modelling change within real world data, during treatment for depression, could inform how these therapies affect symptomatic change and hold potential to better inform shared treatment decision-making.

10.1136/bmjment-2022-300621.supp1 Supplementary data

## Objective

The aims of this study were to (1) identify the direct influence of CBT compared with CfD on symptom change using network intervention analysis^18^ and (2) explore the implications of modelling using either the first appointment in the services (assessment) or the second appointment (first treatment session) as the baseline timepoint and of quantifying symptom change during treatment in a variety of ways: using final scores, difference scores, proportional change and residual scores.

## Methods

### Participants

Routine clinical data were gathered from eight Improving Access to Psychological Therapies (IAPT) services. All were part of the North Central and East London IAPT Service Improvement and Research Network.^19 20^ IAPT services operate as part of a nationwide programme operated by the National Health Service (NHS) to provide evidence-based psychological treatment for depression and anxiety disorders.^21^

Patients are assessed by a clinician to determine their needs and consider the most suitable intervention(s). Patients receive a diagnosis based on International Classification of Diseases, 10th Revision; this represents the focus of treatment agreed on a patient and a clinician. Patients are offered treatment(s) recommended by the National Institute for Health and Care Excellence (NICE) in guidance specific to the patient’s diagnosis.^2^ For less severe depression and anxiety disorders, NICE suggests a stepped-care approach to the delivery of psychological therapies. This means that low-intensity interventions are typically used first, before progressing to more intense treatments if required. For more severe depression, NICE recommends starting with high-intensity face-to-face psychological therapies (such as individual CBT or counselling) in combination with an antidepressant or as a monotherapy. The clinician will outline the interventions that are recommended to the patient and reach a shared decision on a treatment choice appropriate to the person’s clinical needs, considering their preferences. Data from patients who underwent either CBT or CfD treatment for depression (high intensity) and had item-level data available were included in the study. To identify changes due to treatment, only patients who attended five or more treatment sessions were included (see online supplemental eFigure 1 for participant flow).

### Intervention conditions

CfD and CBT were delivered by clinicians with doctoral qualifications in clinical or counselling psychology or with postgraduate diplomas in CBT. Sessions lasted 50–60 min and typically 8–16 sessions were offered. Prior to treatment, patients completed an initial assessment (session 1), and those offered CfD or CBT were placed on a waiting list to start treatment. As such, session 2 represents the first treatment session, typically occurring 4–12 weeks after the assessment session.

For details of the theory underlying these therapies and the competence frameworks, see online supplemental eMethods.

### Outcome measures

IAPT services are mandated to collect sessional outcome data with all patients as well as numerous sociodemographic and treatment-related variables,^22^ and this includes the Patient Health Questionnaire 9-item version (PHQ-9),^23^ a measure of depressive symptoms; and the Generalized Anxiety Disorder Scale 7-item version (GAD-7),^24^ a measure of generalised anxiety disorder symptoms. The items of both measures are used to assess symptom change across treatment. The scores from session 1 (assessment) and session 2 (first treatment session) are used as baseline scores, and the scores in the final treatment session were used as the post-treatment score.

### Statistical analysis

#### Network intervention analysis

Changes scores were estimated for all 16 symptoms of the PHQ-9 and GAD-7. We estimated the residual and difference scores with both session 1 and session 2 as baselines, to account for regression to the mean. Scores were calculated as follows: Difference Score (DS)=postscore−prescore; Final Score (FS)=postscore; Proportional Change (PC)=100*DS/prescore; Residual Score (RP)=postscore−predict value (relationship of prescore–postscore); Residual Change Score (RC)=DS−predict value (relationship of prescore–DS).

Given the potential for topological overlap, we investigated multicollinearity cross-sectionally using the goldbricker function in the networktools package.^25^ There were no node pairs where 75% of correlations were shared with other nodes at any of the timepoints.

Moderated Network Models^26^ were estimated using elastic net regularisation with parameters selected via 10-fold cross-validation, then combining neighbourhood estimates using the AND rule and estimating the linear moderation effects of the interventions. To determine the stability of the estimates (edges and moderating effects), the residual models were refitted using 1000 bootstraps producing bootstrapped sampling distributions of all parameters. Within the network, the associations are conditional on all other variables in the model and the direct effects from the treatment node to the symptoms are the mean change difference in those symptoms between the interventions. The intervention node is binary, where CBT is coded as 1 and CfD as 0. Direct associations are the associations between intervention and changes in individual symptoms, controlling for all other symptoms. We also inspected the three-way interactions (moderation effects) to see how treatment affects the pairwise interactions between the other symptoms.

#### Covariates: propensity score matching

Estimation of the residual models was conducted using the whole sample and a propensity score–matched sample. Propensity score matching was used to control for confounding as the intervention type was not randomly assigned. Matching variables included session 1 item scores (PHQ-9 and GAD-7), gender (male/female), employment status (employed/unemployed), taking psychotropic medication (yes/no), age (continuous), ethnicity (based on UK Census categories: White, Mixed, Asian, Black, Chinese, Other) and baseline functional impairment as measured using the Work and Social Adjustment Scale (Mundt, Marks *et al*, 2002) total score. Propensity score matching was performed using MatchIt package.^27^ Mahalanobis distance matching within the propensity score calliper method (0.25) was used for matching analysis.

#### Total score and symptom change

For comparison purposes, change was modelled between the two interventions on PHQ-9 and GAD-7 sum scores using linear regressions with the final score as the outcome and baseline score as a covariate. This indicates whether the final session score has changed more or less than expected based on the baseline score and the regression equations. This was conducted separately for sessions 1 and 2 as baselines. We also estimated change across each of the 16 individual symptoms (using session 2 as baseline) with false discovery rate (FDR) co rrected p values within both the whole and propensity score–matched samples.

The study has followed the STROBE (Strengthening the Reporting of Observational Studies in Epidemiology) reporting guidelines (see online supplemental eTable 1 for checklist). All materials have been made publicly available via the Open Science Framework and can be accessed at https://osf.io/ak4ev/.

## Findings

### Group characteristics

Total scores on PHQ-9 and GAD-7 were higher at sessions 1 and 2 for the CBT group, and age, ethnicity, gender and number of days between session 1 and 2 (mean difference 6.5 days) differed between the groups (see table 1). There was no evidence of differences between groups on the symptom measures at the final session. Propensity score matching resulted in matching equal numbers of CBT patients to patients in the CfD group (n=3346, 1673 per treatment).

**Table 1:** Sample characteristics and group differences

	CfD	CBT		P value	d/V
	(n=1868)	(n=10 888)			
PHQ-9 total session 1					
Mean (SD)	16.1 (5.81)	16.7 (5.75)		<0.001	−0.12
Median (min, max)	16.0 (0, 27.0)	17.0 (0, 27.0)			
GAD-7 total session 1					
Mean (SD)	13.2 (5.21)	14.1 (4.91)		<0.001	−0.17
Median (min, max)	14.0 (0, 21.0)	15.0 (0, 21.0)			
PHQ-9 total session 2					
Mean (SD)	14.4 (6.37)	15.3 (6.07)		<0.001	−0.15
Median (min, max)	14.0 (0, 27.0)	16.0 (0, 27.0)			
GAD-7 total session 2					
Mean (SD)	12.2 (5.57)	13.2 (5.26)		<0.001	−0.18
Median (min, max)	12.0 (0, 21.0)	14.0 (0, 21.0)			
PHQ-9 total final session					
Mean (SD)	9.23 (6.94)	9.54 (6.80)		0.079	−0.04
Median (min, max)	8.00 (0, 27.0)	8.00 (0, 27.0)			
GAD-7 total final session					
Mean (SD)	8.33 (6.12)	8.26 (5.87)		0.66	0.01
No of sessions: mean (SD)	10.4 (3.9)	10.9 (4.6)		<0.001	0.12
Days between session 1 and session 2	59.9 (49.7)	66.4 (48.9)		<0.001	0.13
Age: mean (SD)	38.5 (13.10)	42.5 (13.5)		<0.001	0.30
Gender				<0.001	0.06
Male	468 (25.1%)	3515 (32.3%)			
Female	1396 (74.7%)	7336 (67.4%)			
Missing/not disclosed	4 (0.2%)	37 (0.3%)			
Ethnicity				<0.001	0.07
Asian	171 (9.2%)	1677 (15.4%)			
Black	232 (12.4%)	1301 (11.9%)			
Chinese	10 (0.5%)	63 (0.6%)			
Mixed	111 (5.9%)	710 (6.5%)			
Other	86 (4.6%)	398 (3.7%)			
White	1210 (64.8%)	6393 (58.7%)			
Missing	48 (2.6%)	346 (3.2%)			

P values and effect sizes reported (Cohen’s d or Cramer’s V).

CBT, cognitive–behavioural therapy; CfD, counselling for depression; GAD-7, Generalized Anxiety Disorder Scale 7-item version; PHQ-9, Patient Health Questionnaire 9-item version.

### Network intervention analysis

**Figure 1:**
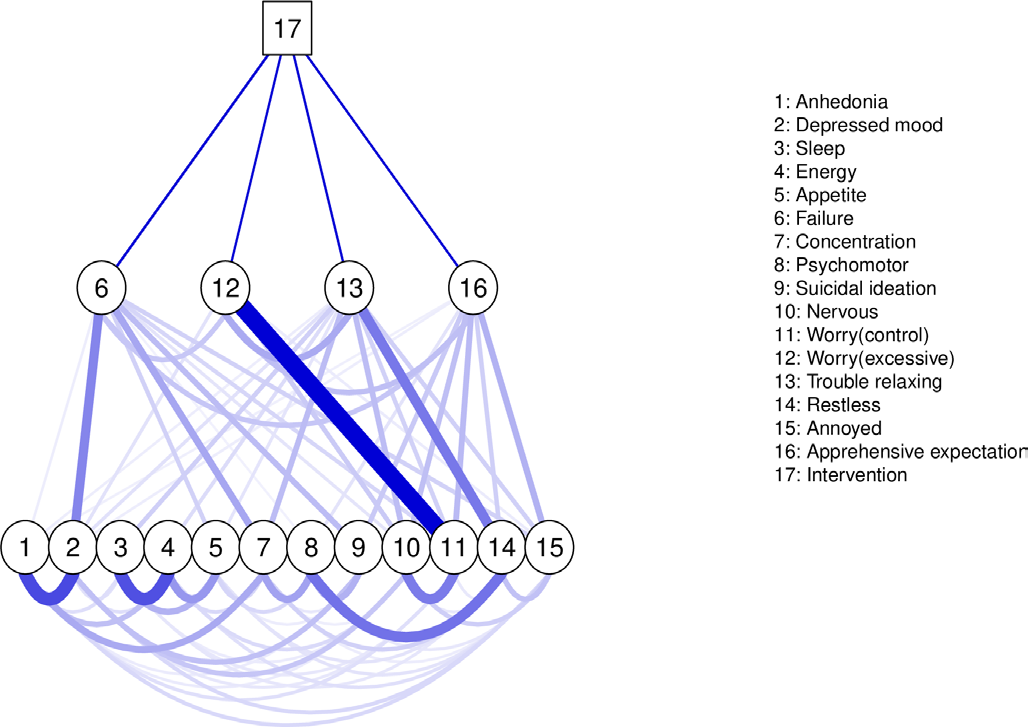
Network plot (RCX2). This represents the propensity-matched models which were virtually identical. The network includes intervention (CBT or CfD) as a square node and items from the PHQ-9 and GAD-7. The thickness and saturation of the edges between symptoms are proportional to the strength of the association. Within the mixed graphical model, the inclusion of the intervention node (CBT coded as 1 and CfD as 0) allows us to explore moderation effects, identifying symptoms that are uniquely influenced by the intervention type, thereby demarcating intervention-specific effects with the network. Edges between intervention and a symptom indicate a larger direct item-specific effect for one of the interventions, but direct effects that are shared by both interventions will not be included into the network model. This direct effect may account for the spread throughout the network and indicate likely pathways through which an intervention may influence symptoms. The edges between the intervention node and symptoms are direct associations—the heatmap below indicates the strength and direction of these associations. CBT, cognitive–behavioural therapy; CfD, counselling for depression; GAD-7, Generalized Anxiety Disorder Scale 7-item version; PHQ-9, Patient Health Questionnaire 9-item version; RC, Residual Score (change score–baseline); X, propensity score–matched samples.

**Figure 2:**
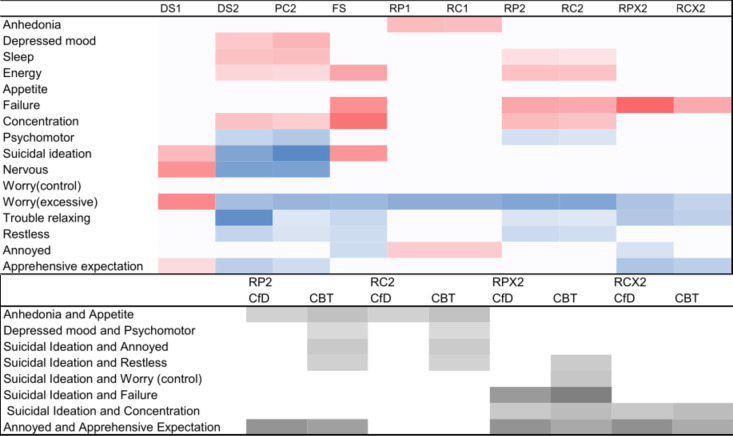
(Top) Heat map of direct associations for each model. The heatmap displays the direct associations between symptoms and intervention type obtained using the different methods of calculating change and against different baseline timepoints. Colour scale: darker=stronger, with blue reflecting direct associations with CBT and red reflecting direct association with CfD. In the headings, the number refers to the baseline used (ie, session 1 or 2), DS, Difference Score; FS, Final Score only; PC, Proportional Change; RP, Residual Score (post score–baseline); RC, Residual Score (change score–baseline); X, propensity score–matched samples. (bottom) The influence of the type of intervention on symptom-to-symptom interactions. The values represent the presence and strength of the influence for the associations that differentiate the interventions. Colour scale: darker=stronger. CBT, cognitive–behavioural therapy; CfD, counselling for depression.

The propensity score model is plotted in figure 1 (all models are plotted in online supplemental eFigure 2), and the direct associations are specified in figure 2. Most edges were reliably estimated and included in all or nearly all of the 1000 bootstrapped samples (online supplemental eFigures 2 and 3).

Results using the difference score with session 1 as the baseline were different than other change models (eg, correlation between matrices DS and RC1, r>0.48), with the direct associations negatively correlated will all other estimates, including modelling the difference score with session 2 as the baseline, r=−0.60. Direct associations found with residual score models using the session 1 baseline were different from those found using session 2 data. The associations found when using session 2 as the baseline were consistent whether using the final score or residual score outcome (r>0.98).

The whole sample residual models using the session 2 baseline were similar (r>0.99), and similar to the propensity score–matched models (r>0.98). Fewer direct associations were identified in the propensity score–matched sample using the residual change score outcomes. In these models, using the session 2 baseline, there was consistency across four items identified as having direct associations, three positively associated with CBT and one positively associated with CfD. Across the propensity score–matched models, there was a larger change of scores on thoughts of being a failure with CfD (RCX2: 0.03) and a larger effect on excessive worry (RCX2: 0.02), troubling relaxing (RCX2: 0.02) and apprehensive expectation (RCX2: 0.02) with CBT.

When looking at the influence of treatment on symptom-to-symptom interactions (figure 2), there was less consistency between models. While there was consistency between residual models within samples, there was very little between samples (whole and propensity score matched).

Within the whole sample, there was evidence of stronger related change between anhedonia and appetite during CBT than CfD (CBT: 0.05, CfD: 0.03). Further, the CBT group showed an associated change between suicidal ideation and restlessness (0.03), suicidal ideation and being easily annoyed/irritated (0.04) and between depressed mood and psychomotor disturbance (0.01); these were absent for the CfD group.

Between the propensity score–matched models, only two effects were identified in both models: the CfD group showed a stronger related change between feeling annoyed and apprehensive expectation (CBT: 0.09, CfD: 0.13). There was also a difference between groups on the associated change between suicidal ideation and concentration (CBT: 0.06, CfD: 0.04), with the CBT group displaying stronger associated change than the CfD group. Given the difference between interventions on the number of sessions attended, we controlled for the number of sessions within the RCX2 model. This did not alter any of the direct or indirect effects (see online supplemental eFigure 4). Within the discussion, only interactions observed across both propensity score models are interpreted.

### Total score and symptom change

Within the whole sample, there was a greater degree of change in anxiety but not depression during CBT than CfD. This difference was larger for the final GAD-7 score when controlling for session 2 scores: F(1,12753)=24.255, p<0.001, ω_p_²=0.002, estimated marginal means±SE (CBT: 8.28 (0.05), CfD: 8.98 (0.13)) than when controlling for session 1 scores: F(1,12753)=17.94, p<0.001, ω_p_²=0.002 (CBT: 8.29 (0.06), CfD 8.9 (0.14)). There was no evidence of a difference between groups for the final PHQ-9 total score when controlling for session 1 PHQ-9 scores: F(1,12753)=1.3, p=0.254 (CBT: 9.58 (0.06), CfD: 9.77 (0.16) or session 2 scores: F(1,12753)=4.385, p=0.036 (CBT: 9.56 (0.06), CfD: 9.90 (0.15)). Within the propensity score–matched sample, there was a greater degree of change in both anxiety and depression during CBT than CfD when controlling for the session 2 score, PHQ total score: F(1,3443)=6.836, p<0.009 (CBT: 8.89 (0.14), CfD: 9.40 (0.14), and GAD-7 total score: F(1,3443)=18.35, p<0.001, ω_p_²=0.005 (CBT: 7.72 (0.13), CfD:8.47 (0.12)).

**Figure 3:**
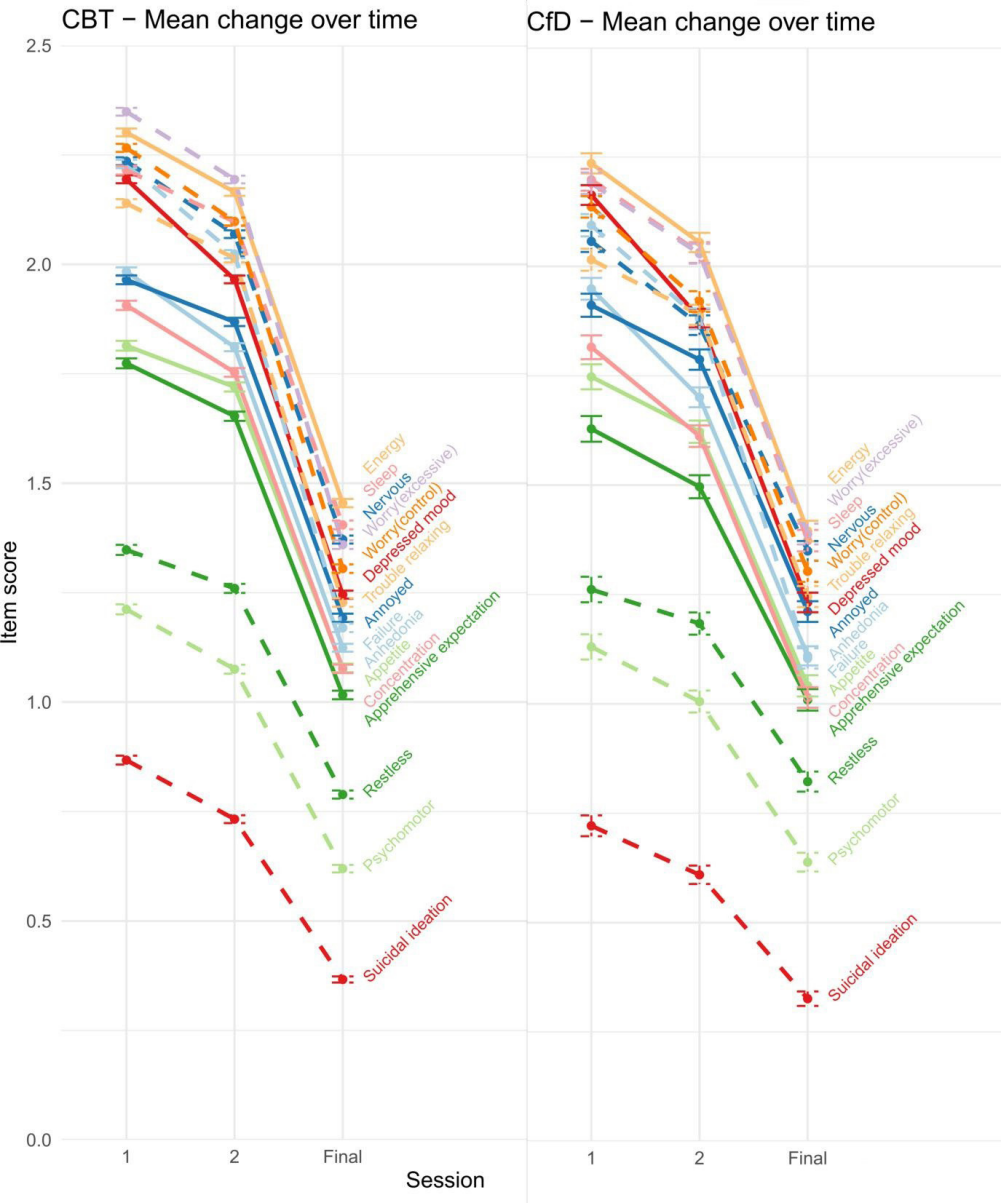
Mean change and SE for each symptom at session 1 and 2 (baseline measures) and the final session of treatment.

Symptom change is plotted in figure 3. After correcting for FDR, there was evidence that all GAD-7 symptoms and psychomotor disturbance were lower at end point for CBT than CfD (online supplemental eTable 2). Within the propensity score–matched samples, anhedonia, depressed mood, suicidal ideation and all the GAD-7 symptoms were lower at end point for CBT than CfD.

## Conclusions

This study investigated differences in symptom-specific effects of CBT and CfD, and the impact of modelling symptom changes in a variety of commonly used ways for adults with depression treated in primary/community care psychological therapy services. We found that CBT for depression may work by directly affecting excessive worry, trouble relaxing and apprehensive expectation, while CfD may work by affecting thoughts of being a failure. These effects were specific to the type of treatment, that is, they were not shared effects (where both interventions similarly affect symptoms this is not visualised) or indirect effects of changes in other symptoms influenced by the treatments. There were also treatment-specific effects on symptom-to-symptom interactions. CfD had a stronger influence on the associated change between feeling annoyed and apprehensive expectation than CBT. The associated change between suicidal ideation and concentration was greater for CBT than CfD.

We found variability in the results obtained from different ways of measuring change. There was little consistency in the results between using session 1 and session 2 as a baseline. This is important because many observational studies and clinicians use pre–post change in a symptom measure score as their primary outcome. Further, within treatment settings, there can be a period (weeks to months) between initial assessment (session 1) and commencing treatment (session 2). Hence, session 2 appears to be a more appropriate baseline for measuring treatment-related symptom change. Differences between the whole and propensity score–matched samples would suggest that there is an influence of covariates, but it is less evident when estimating direct associations, although propensity score matching cannot fully redress selection biases or confounding given the potential influence of unmeasured variables.^28^ The difference score and proportional change models produced inconsistent results; however, the final score model (a simple method) and residual score approaches were consistent. This echoes the established but rarely adhered to methodology of regressing the second baseline measurement (baseline) on the postscore or difference score where a residual score for each participant can be modelled within the network.^29^ Although established for clinical trials, this also appears to fit for observational data in naturalistic settings.

The results provide evidence to elucidate how these therapies may work. For example, compared with CBT, CfD was directly associated with a change in the thoughts of being a failure. CfD also demonstrated a greater associated change between feeling annoyed and apprehensive expectation (feeling afraid that something bad will happen) than CBT. This fits with the theoretical underpinnings of CfD targeting the development of self-concept and conditions of worth and their link to emotional processes.^30^ CBT encompasses a number of approaches to tackling depression as most of which also target beliefs about the self; however, it appears that this effect may not be as direct as it was in CfD. It might be that in the CBT delivery there was a greater focus on altering ruminative thinking processes than the content of negative thoughts and self-beliefs themselves.^31^ For both treatments, self-beliefs may represent an important target as we found an indirect effect of treatments on depressed mood via thoughts of being a failure.

CBT for depression was uniquely associated with changes in symptoms associated with anxiety. Some of the observed symptom effects could be considered mechanistic (reflecting an underlying physiological, neurobiological or functional mechanism) others are more descriptive.^32^ The changes in excessive worry and apprehensive expectation were both uniquely associated with CBT and, as another form of repetitive negative thinking (like rumination), have been identified as a transdiagnostic mechanism and treatment target.^33^ Excessive worry has a strong temporal influence on the change in other symptoms during psychotherapy,^10^ and CBT has been found to have a moderate effect on repetitive negative thinking.^34^ CBT was also directly associated with trouble relaxing. Trouble relaxing has been identified as a central symptom within remission networks following CBT^35^ and as a bridge between symptoms of anxiety and depression.^36^ There is some evidence that these symptoms are associated with experiential avoidance so CBT might be bringing about symptom change by tackling this process.^37^

There was a stronger associated change between suicidal ideation and concentration for CBT than CfD. Within this sample, we cannot identify temporal precedence. However, in a dynamic network model of change during psychotherapy, temporal influence was stronger for concentration on suicidal ideation than the other way around.^10^ Concentration has been identified as a central symptom in a relapse network^35^ and maybe reflective of poor meta-cognitive capacity to regulate impulsive tendencies to harm oneself.^38^ Although not evidenced in both models, there was an indication that CBT may be associated with a change between suicidal ideation and several symptoms (restlessness, feelings of failure and controllability of worry) suggesting indirect pathways through which CBT may reduce suicidal ideation.

### Limitations

We attempted to balance groups on observed covariates, but they may have differed on important, unmeasured confounders such as those related to aspects of severity,^39 40^ to sociodemographics or socioeconomic factors,^15 41^ and as such the differences observed may be due to external factors. There are other selection variables and mechanisms of interest to measure when comparing these treatment approaches. For example, previous experiences of treatment, where those who received CfD may have previously had CBT, adherence to treatment (fidelity and engagement) or therapeutic alliance which has been shown to influence change.^42^ The PHQ-9 and GAD-7 cover core symptoms; however, there are many other symptoms of depression and anxiety^7^ that are relevant to understanding the mechanisms of change within these treatments. Second, the study measures change between two timepoints, dynamic processes of change are more complex^10^ and the temporal relationship in respect of each treatment is unknown and would be worth exploring in future research. Third, the analysis represents the largest network comparison of psychological treatments to-date; however, at the individual level, knowledge of individual symptoms alone might not be sufficient to inform clinical decisions, and it may not lead to better prognostic predictions or make it easier to select between generally similar treatment types.^43^ This is not to say the findings are not clinically meaningful, as they can be important when implementing decision-making at the population level (eg, around treatment selection and outcome measurement) potentially leading to improved recovery rates on a mass level. Finally, this study provides a methodological illustration of the different results that emerge from modelling decisions rather than a statistical comparison of models. While these findings illustrate issues with difference scores that have been well established within the RCT literature (see online supplemental eMethod), a simulation study would be required to assess the robustness of a given model in various scenarios. Equally, while the study employs a large sample, increasing the accuracy of parameter estimates, replication in an independent sample would be required. These may inform the determination of treatment outcomes in routine clinical care and future observational studies alike.

## Clinical implications

It is important to understand how interventions work so that more effective and efficient treatments can be developed, and so that interventions can be more acceptable to patients. This study suggests that as CBT was uniquely associated with changes in symptoms associated with anxiety it may be better suited to those with anxiety symptoms comorbid to their depression.

The study also highlights methodological considerations. When assessing change, the baseline should be the first therapy session (or second session) not the pretreatment assessment. This will address potential sources of bias such as regression to the mean. When calculating change, residual change scores should be preferred over difference score methods.

## Data Availability

Data are available on reasonable request. The raw data are not publicly available due to the containing information that could compromise participant privacy/consent. The raw data that support the findings of this study are available on request from JEJB subject to appropriate permissions from the custodians of the data restrictions apply to the availability of these data, which were used under license for the current study. The data were provided by the IAPT services for evaluation as part of a wider service improvement project conducted in accordance with the procedures of the host institution and the NHS Trusts which operate the IAPT services (project reference: 00519-IAPT).
