# Supplementary material for: Symptom-specific effects of counselling for depression compared to cognitive–behavioural therapy
Source: BMJ Ment Health. 2023 Feb 15;26(1):e300621. doi: 10.1136/bmjment-2022-300621 (PMC10035778; doi:10.1136/bmjment-2022-300621)
Supplement: Supplementary data [file bmjment-2022-300621supp001.pdf]

Supplementary Materials

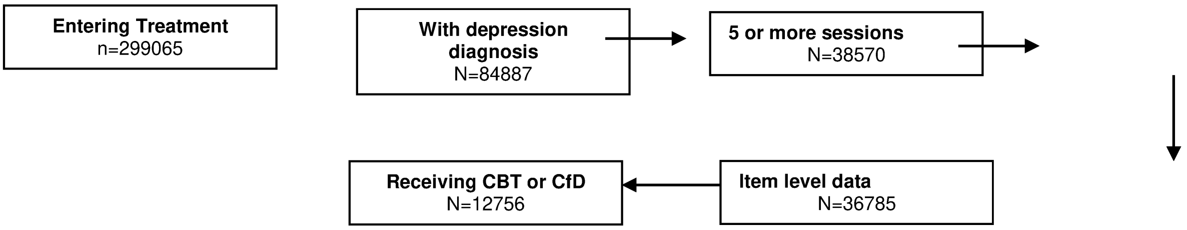

eFigure 1. Participant Flow with Reasons for Inclusion

eTable 1 STROBE Statement—Checklist of items that should be included in reports of ***cohort studies***

EMethods

The implications of modelling choices when calculating change

There are several numerical methods to measure change, and comparisons of approaches are not new [1]. Four approaches in the literature are common, difference (or gain) scores, percentage (or proportional) change, post-score only, and residual change. There's a general consensus that calculating residual change where the final score is the outcome while adjusting for the baseline score is the most efficient approach [2–4]. However, in both research and clinical practice, differences scores are most used due to ease of calculation and simple, if misleading, interpretation. These different approaches can lead to different results (eg, Lord's paradox) [1]. The difference score does not adjust for baseline imbalance, and indeed these may be reversed [5] – patients with high scores at baseline generally improve more than those with low scores: (where higher scores indicate greater severity). Although difference scores appear intuitively interpretable, where the measure is ordinal (as in most psychological measures), the difference score will no longer be ordinal [6]. Measuring within-person change will also be affected by regression to the mean and measurement error. In addition, in comparison to RCTs, within clinical practice, allocation to an intervention will be informed by patient preference as well as clinical decision making, which in turn and the person's preference, the decision making will partly be informed by symptomatology. This introduces a selection bias, where future measurements will be closer to the person's true mean [7]. A preferred method is to use the final score as the outcome and baseline as

covariate, although this method is not impervious to bias, particularly when there are pretreatment differences [4]. An alternative approach is to incorporate the change score as the outcome and baseline score as covariate, the benefit of which is purported to assess whether change occurred in each group [8]. How change is calculated can lead to inaccurate estimation of association, and are unlikely to result lead to the same result [9]. While comparisons have been studied [10] this has not be investigated within the network literature.

Details of the theory underlying these therapies and the competence frameworks they are based on.

CfD is a manualized form of psychological therapy derived from the humanistic competence framework [11] using therapy manuals from randomised controlled trials. The specific area of humanistic practice on which the CfD competences are based is termed person-centred/experiential therapy [12,13] and also integrates aspects of emotion-focused therapy [14]. This modality targets the emotional problems underlying depression along with intrapersonal processes (such as excessive self-criticism) that often maintain depressed mood. The therapy aims to help patients contact underlying feelings, make sense of them, and reflect on the new meanings which emerge.

High Intensity CBT involves structured, protocol-driven, disorder-specific interventions in line with set competencies [15]. Psychotherapists are trained based on a national curriculum [16]. Second wave CBT approaches to depression combine behavioural and cognitive approaches to modify biases in information processing and avoidance behaviours (e.g. Beck, Rush, Shaw, & Emery, 1979).

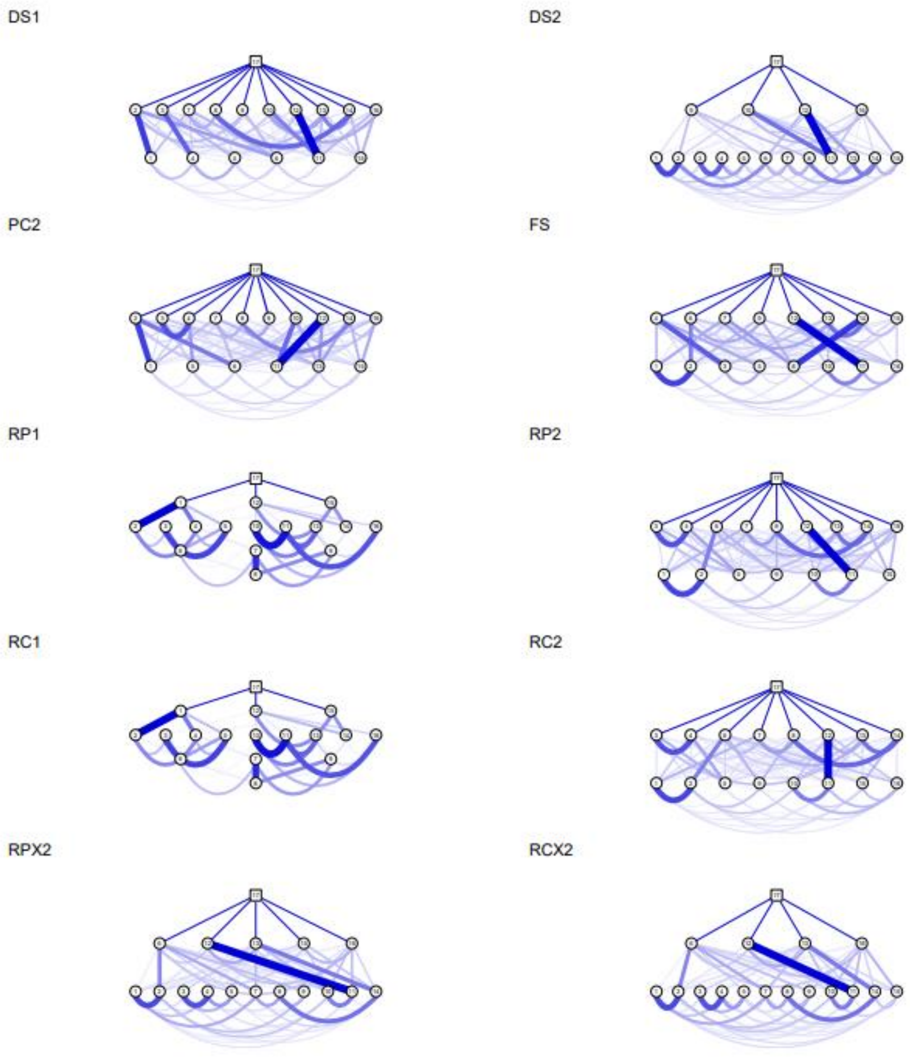

eFigure 2: The networks correspond to the different approaches to measuring change. The networks include intervention (CBT or CfD) as a square node and items from the PHQ-9 and GAD-7. The thickness and saturation of the edges between symptoms is proportional to the strength of the association. The edges between the intervention node and symptoms are direct associations – the heatmap below indicates the strength and direction of these associations.

Symptom	Whole Sample		Propensity Score Matched	
	Statistic	EMM (SE) CBT, CfD	Statistic	EMM (SE) CBT, CfD
Anhedonia	$F(1,12753) = 1.394$, $p=0.238$	-	$F(1,3443) = 5.266$, $p=0.036$*, $\omega_p^2 = 0.001$	1.05(0.02),1.12(0.02)
Depressed mood	$F(1,12753) = 3.182$, $p=0.132$	-	$F(1,3443) = 5.226$, $p=0.030$*, $\omega_p^2 = 0.001$	1.17(0.02),1.24(0.02)
Sleep	$F(1,12753) = 0.004$, $p=0.95$	-	$F(1,3443) = 0.430$, $p=0.468$	
Energy	$F(1,12753) = 0.232$, $p=0.63$	-	$F(1,3443) = 0.908$, $p=0.406$	
Appetite	$F(1,12753) = 0.010$, $p=0.92$	-	$F(1,3443) = 2.903$, $p=0.175$	
Failure	$F(1,12753) = 0.011$, $p=0.92$	-	$F(1,3443) = 1.217$, $p=0.325$	
Concentration	$F(1,12753) = 0.109$, $p=0.741$	-	$F(1,3443) = 2.786$, $p=0.163$	
Psychomotor	$F(1,12753) = 11.026$, $p=0.003$*, $\omega_p^2 = 0.001$	0.63(0.01),0.70(0.02)	$F(1,3443) = 2.902$, $p=0.133$	
Suicidal ideation	$F(1,12753) = 0.652$, $p=0.419$		$F(1,3443) = 5.266$, $p=0.036$*, $\omega_p^2 = 0.001$	0.29(0.02),0.33(0.02)
Nervous	$F(1,12753) = 7.124$, $p=0.008$*, $\omega_p^2 = 0.001$	1.37(0.01),1.43(0.02)	$F(1,3443) = 4.428$, $p=0.043$*, $\omega_p^2 = 0.001$	1.30(0.02),1.37(0.02)
Worry(control)	$F(1,12753) = 9.653$, $p=0.005$*, $\omega_p^2 = 0.001$	1.31(0.01),1.39(0.02)	$F(1,3443) = 7.367$, $p<0.008$*, $\omega_p^2 = 0.001$	1.23(0.02),1.32(0.02)
Worry(excessive)	$F(1,12753) = 17.087$, $p<0.001$*, $\omega_p^2 = 0.001$	1.36(0.01),1.48(0.02)	$F(1,3443) = 13.151$, $p<0.002$*, $\omega_p^2 = 0.004$	1.35(0.02),1.38(0.02)
Trouble relaxing	$F(1,12753) = 11.115$, $p=0.003$*,, $\omega_p^2 = 0.001$	1.23(0.01),1.32(0.02)	$F(1,3443) = 13.445$, $p<0.002$*, $\omega_p^2 = 0.004$	1.38(0.02),1.41(0.02)
Restless	$F(1,12753) = 12.991$, $p=0.003$*, $\omega_p^2 = 0.001$	0.80(0.01),0.89(0.02)	$F(1,3443) = 8.243$, $p<0.008$*, $\omega_p^2 = 0.002$	1.00(0.02),1.06(0.02)
Annoyed	$F(1,12753) = 5.759$, $p=0.032$*, $\omega_p^2 < 0.001$	1.19(0.01),1.26(0.02)	$F(1,3443) = 9.011$, $p=0.008$* $\omega_p^2 = 0.002$	1.11(0.02),1.12(0.02)
Apprehensive expectation	$F(1,12753) = 11.511$, $p=0.003$*, $\omega_p^2 = 0.001$	1.02(0.01),1.11(0.02)	$F(1,3443) = 12.362$, $p=0.002$*, $\omega_p^2 = 0.004$	1.01(0.02),1.04(0.02)

eTable 2: Raw symptom change statistics. P-values corrected for FDR. EMM= Estimated Marginal Means

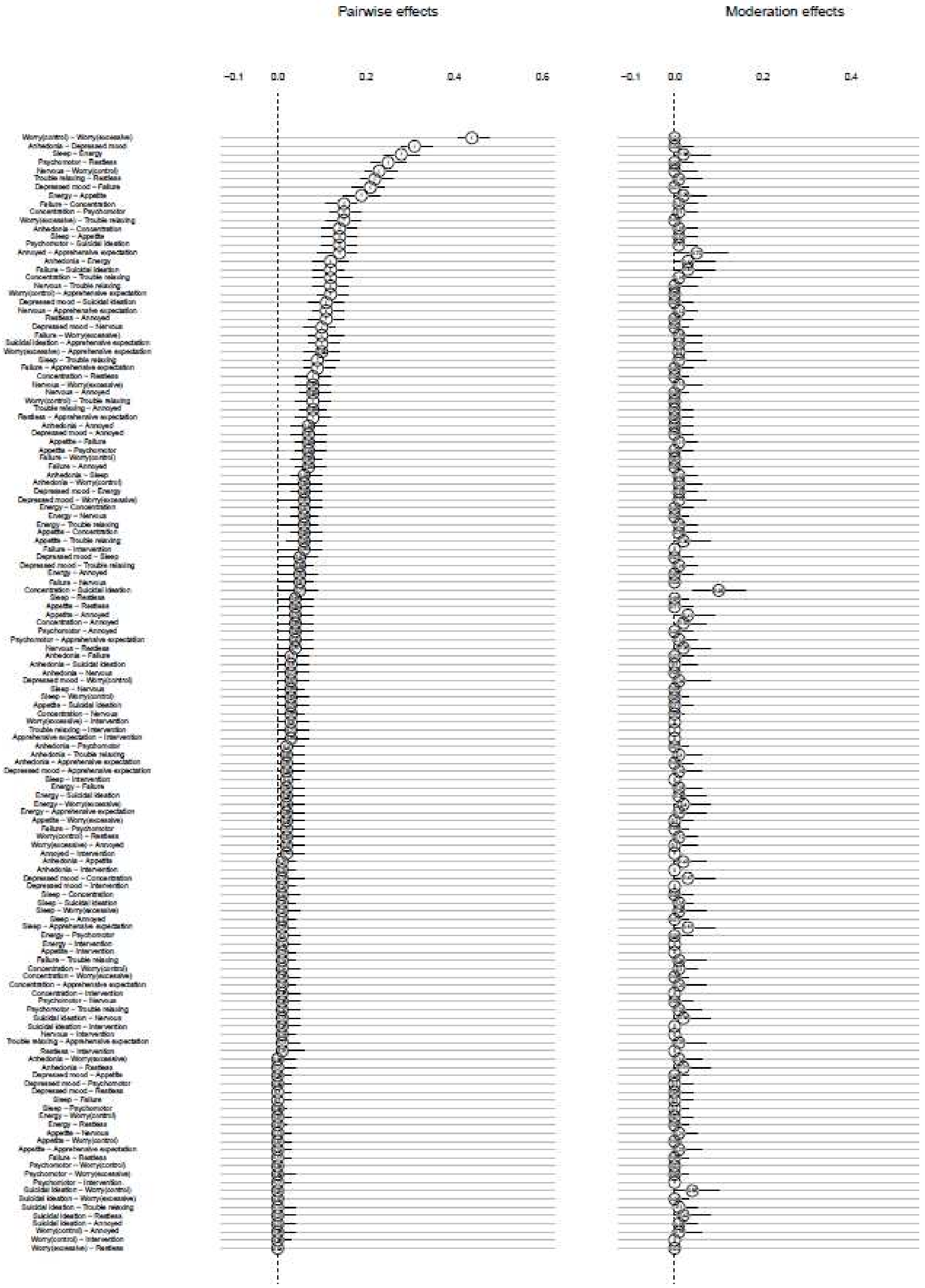

eFigure 2: Bootstrapped stability plot (RCX2). A higher resolution image can be found here: https://osf.io/j7sve

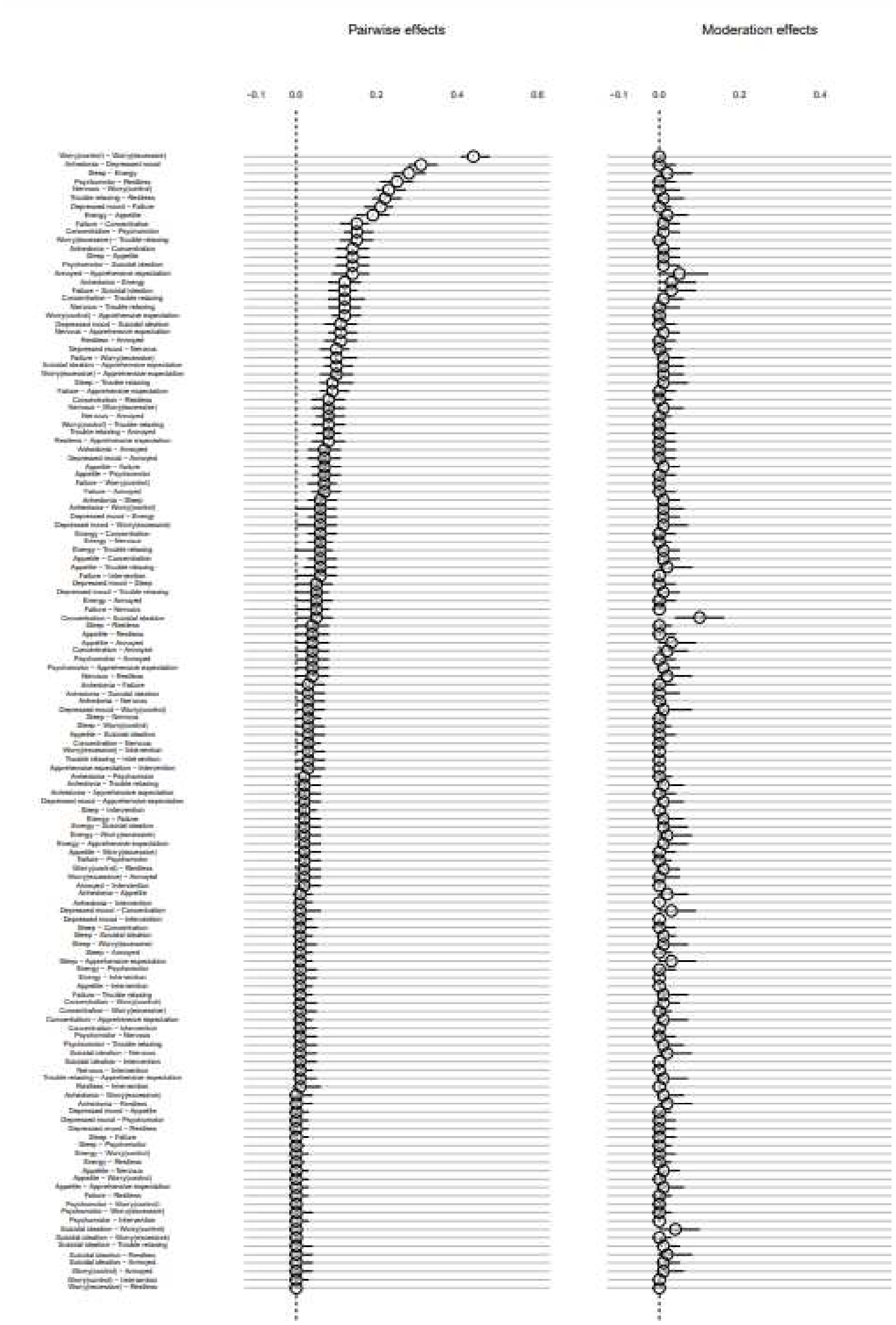

eFigure 3: Bootstrapped stability plots (RPX2) A higher resolution image can be found here: https://osf.io/uer5w

RC2x including number of sessions

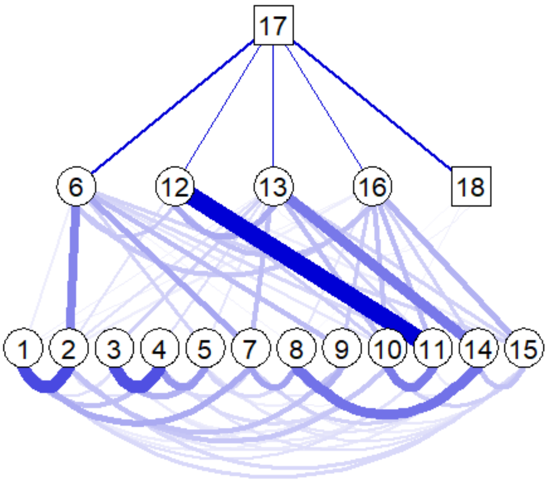

eFigure 4. Sensitivity Analysis including number of sessions (18) with the residual score network model using session 2 as prescore, within the propensity score matched samples. The number of sessions was associated with change anhedonia (8), sleep (10) and failure (13), but there was no differential effects between interventions and the inclusion of the number of sessions didn't change any of the direct or indirect effects (near perfect correlation between matrices r = 0.9997).

References

1 Lord FM. A paradox in the interpretation of group comparisons. *Psychol Bull* 1967; **68**: 304–5.

2 Vickers AJ. The use of percentage change from baseline as an outcome in a controlled trial is statistically inefficient: a simulation study. *BMC Med Res Methodol* 2001; **1**: 6.

3 Bland JM, Altman DG. Comparisons against baseline within randomised groups are often used and can be highly misleading. *Trials* 2011; **12**: 264.

4 Senn S. Change from baseline and analysis of covariance revisited. *Stat Med* 2006; **25**: 4334–44.

5 Vickers AJ, Altman D. Statistics Notes: Analysing controlled trials with baseline and follow up measurements. *BMJ* 2001; **323**: 1123–4.

6 Harrell F. Change Scores. 2018. https://www.fharrell.com/tag/change-scores/.

7 Barnett AG, van der Pols JC, Dobson AJ. Regression to the mean: What it is and how to deal with it. *Int J Epidemiol* 2005; **34**: 215–20.

8 Laird N. Further Comparative Analyses of Pretest-Posttest Research Designs. *Am Stat* 1983; **37**: 329–30.

9 Nickerson CA, Brown NJL. Simpson's Paradox is suppression, but Lord's Paradox is neither:

Clarification of and correction to Tu, Gunnell, and Gilthorpe (2008). *Emerg Themes Epidemiol* 2019; **16**: 1–11.

10 O'Connell NS, Dai L, Jiang Y, *et al.* Methods for Analysis of Pre-Post Data in Clinical Research: A Comparison of Five Common Methods. *J Biom Biostat* 2017; **08**. DOI:10.4172/2155-6180.1000334.

11 Roth AD, Hill A, Pilling S. The competences required to deliver effective Humanistic Psychological Therapies. London: University College London, 2009.

12 Mearns D, Thorne B. Person-centred counselling in action, 3rd edn. London: Sage, 2007.

13 Elliott R, Watson JC, Goldman RN, Greenberg LS. Learning emotionfocused therapy: The process-experiential approach to change. Washington DC: APA, 2004.

14 Greenberg L, Watson J. Experiential Therapy of Depression: Differential Effects of Client-Centered Relationship Conditions and Process Experiential Interventions. *Psychother Res* 1998; **8**: 210–24.

15 Roth AD, Pilling S. Using an Evidence-Based Methodology to Identify the Competences Required to Deliver Effective Cognitive and Behavioural Therapy for Depression and Anxiety Disorders. *Behav Cogn Psychother* 2008; **36**: 129–47.

16 Department of Health. National curriculum for Cognitive Behavioural Therapy courses, Second edi. Kings College London, 2011.

17 Beck AT, Rush AJ, Shaw BF, Emery G. Cognitive therapy of depression. New York: Guilford Press, 1979.
